# Restricted diffusion of the callosal splenium is highly specific for seizures in neonates

**DOI:** 10.1186/s12883-022-02984-9

**Published:** 2022-12-06

**Authors:** Linda Nguyen, Dillon Y. Chen, Daniel N. Vinocur, Jeffrey J. Gold

**Affiliations:** 1 Department of Neurosciences, University of California San Diego, San Diego, California USA; 2 Division of Neurology, Rady Children’s Hospital San Diego, San Diego, California USA; 3 Department of Radiology, Rady Children’s Hospital San Diego, San Diego, California USA

**Keywords:** Neonatal, Neonatal seizure, Splenium, Corpus callosum, Hypoxic ischemic encephalopathy

## Abstract

**Background:**

To determine whether restricted diffusion of the callosal splenium is specific for seizure activity in neonates.

**Methods:**

We performed a retrospective chart review of 123 neonates who had a diagnosis of hypoxic ischemic encephalopathy (HIE) who underwent therapeutic cooling and had magnetic resonance imaging (MRI) within the first 10 days of life. The regions examined for injury include the callosal splenium, cortex, deep gray matter, and subcortical white matter. Neurodevelopmental outcomes were secondarily assessed using the Bayley Scales of Infant Development at 12 to 18 months of age and > 18 months of age. APGAR scores and pH, two important markers of hypoxia/ischemia and encephalopathy, were also analyzed in relation to these outcomes.

**Results:**

Approximately 41% of the neonates had at least one abnormal region on brain MRI, and 21% had abnormal signal in the splenium. Clinical and/or electrographic seizures were documented in 32%. Changes in the splenium had a sensitivity of 54%, specificity of 94%, and positive predictive value of 81% for seizure presence. The presence of seizures and splenium lesion was associated poor developmental outcomes at 12 to 18 months of age. APGAR scores at 10 minutes, but not lowest pH was associated with splenial changes.

**Conclusions:**

Restricted diffusion of the callosal splenium is specific for recent seizures in neonates with HIE. Seizures and splenial lesion represent risk factors for poor neurodevelopmental outcomes. Child neurologists and neonatologists should consider splenial signal abnormality in their assessment of neonates at risk for seizures and counsel families about likely outcomes accordingly.

## Background

The corpus callosum (CC) is the largest white matter tract in the brain, composed of more than 200 million axons [1]. The thickest, most posterior aspect of the CC is the splenium. On magnetic resonance imaging (MRI), restricted diffusion in the splenium has been shown to be associated with recent seizures in adults with certain types of epilepsy [2–6]. In neonates, this has been suggested in isolated case reports [7–9], but no systematic study has been carried out to investigate the utility of this finding. We conducted a retrospective chart review to evaluate whether MRI changes in the splenium were associated with the presence of recent seizure in neonates. In addition, we examined whether splenial signal changes and seizures were associated with poor outcomes. We studied a consecutive series of neonates who were diagnosed with hypoxic ischemic encephalopathy (HIE) and underwent therapeutic cooling. This was an ideal population to isolate the impact of seizures on MRI changes in the splenium because these neonates received a standard amount of video electroencephalogram (EEG) recording and obtained MRI in or around the first week of life.

## Methods

### Patients

We retrospectively reviewed the medical charts of neonates admitted after birth to a level IV neonatal intensive care unit (NICU) at Rady Children’s Hospital (RCH) between January 2015 and December 2018 who were diagnosed with HIE. Neonates qualified for therapeutic cooling according to the most recent Cochrane Review [10]: (1) newborns ≥ 35 weeks gestation; (2) evidence of peripartum asphyxia (at least one of the following): APGAR ≤ 5 at 10 minutes, mechanical ventilation or resuscitation at 10 min, and/or cord pH < 7.1 or an arterial pH < 7.1 or base deficit ≥ 12 within 60 min; (3) evidence of encephalopathy according to Sarnat staging; and (4) no major congenital abnormalities recognizable at birth. Neonates in our study were identified by (a) searching RCH’s entries into the Children’s Hospital Neonatal Database and (b) reviewing neonatal neurology consultation service records. Data from all patients were de-identified. The need for informed consent was waived by the ethics committee/Institutional Review Board of the University of California, San Diego School of Medicine and Rady Children’s Hospital because of the retrospective nature of the study.

### Inclusion and exclusion criteria

Neonates with the diagnosis of HIE, therapeutic cooling performed, and MRI obtained within the first 10 days of life were included. Neonates whom MRI studies were obtained beyond 10 days of life were excluded to prevent the phenomenon of “pseudo-normalization” to confound our findings [11]. Neonates without MRI studies, lacking documentation of HIE, unqualified for therapeutic cooling, or transferred to our facility later than day of life 1 were excluded as well.

### Data collection

Data were extracted from the medical charts and included: gestational age, birth weight, method of delivery, acute events surrounding delivery (non-reassuring fetal heart tones, pre-eclampsia, placental abruption, uterine rupture, cord prolapse, nuchal cord, meconium stain, and chorioamnionitis), intubation, and chest compression. APGAR scores were taken at 1, 5, and 10 minutes. Cord blood gases and the earliest venous and arterial blood gases were collected from the NICU admitting note or first neurology consult note. Sarnat staging (mild, moderate, or severe) was collected from the first neurology note because it usually had the first and only documented Sarnat staging score.

Data on presence or absence of seizure, EEG report and anti-epileptic therapies given during NICU admission were collected. Neonates who underwent therapeutic cooling were standardly placed on video EEG on admission and continued for at least 24 hours after re-warming. A full neonatal montage recording was made with the following channels FP1-T3, T3-O1, FP1-C3, C3-O1, FP2-T4, T4-O2, FP2-C4, C4-O2, T3-C3, C3-Cz, Cz-C4, C4-T4, FP1-O1, FP2-O2, C3-C4, and EKG. Neonates with clinical, electrographic, and/or clinical with electrographic correlate seizures were included in the seizure group. Clinical seizure is defined as abnormal movements thought to be seizure and subsequent treated with an anti-epileptic prior to the neonate being hooked up to EEG. Seizure burden was categorized by seizure frequency (none, 1 seizure, 2 or more seizures, or status epilepticus, which is defined as > 30 min of seizure within any 1-hour epoch) and number of anti-epileptics used (none, 1, 2 or more not including midazolam drip, or includes midazolam drip). The most abnormal EEG background was determine based on the EEG report in the first 24 hours, categorized as normal, mild/moderately abnormal, or severely abnormal (markedly excessive discontinuity, burst suppression, gross interhemispheric asynchrony, or extreme low voltage).

For splenial changes, all the MRI images were blindly reviewed by a pediatric neuroradiologist (DNV) for the presence or absence of restricted diffusion in the splenium. For the presence or absence of any abnormality in the cortical ribbon, deep gray matter, or subcortical white matter, data was obtained from the MRI report. The MRI brain (without contrast or with and without contrast) was obtained as early as 24 hours after re-warming. If a patient had undergone more than one MRI examination, the earliest scan was utilized for the analysis.

As part of our routine clinical care for neonates with HIE, neurodevelopmental testing using the Bayley Scales of Infant Development, Third Edition (BSID-III) was performed by trained medical providers upon follow up in the high-risk infant clinic. This specialty clinic evaluated the growth and development of infants at risk for neurologic problems or developmental delays after discharge from the NICU. Children are usually seen first around 6 months of age and then 1 to 2 times per year after until 3 years of age. For further analysis, scores obtained at 12 to 18 months of age and > 18 months of age were used. A moderate delay was defined as a BSID-III score 1 to 2 standard deviations below the norm, i.e. lowest composite score of 70 to 84 in any of three domains (cognitive, language, and motor). Severe delay was defined as a BSID-III score greater than 2 standard deviations below the norm, i.e. less than 70 on any of the three tested domains or a complete inability to assign a score due to severe mental deficiency.

### Statistical analysis

Data were summarized with counts (percentages) for categorical variables and as the mean ± standard deviation for continuous variables. Sample characteristics, including gestational age at birth, sex, and day of life at the time of MRI, were investigated using descriptive analyses. The value of MRI findings with respect to seizure presence was expressed as sensitivity, specificity, positive predictive value (PPV), negative predictive value (NPV), positive likelihood ratio (LR) and negative LR. To determine if there was a relationship between seizure, pH level, APGAR score, MRI abnormality and neurodevelopmental outcome, the following groups were formed: (a) presence or absence of seizure, (b) pH < 7.1 or ≥ 7.1, and (b) APGAR score ≤ 5 or > 5 at 5 and 10 minutes. Group differences were examined using Chi-square analysis or unpaired t-test using SPSS Statistics. Significance was set at *P* ≤ 0.05.

## Results

During the study period, 188 neonates with HIE were identified. Twenty-one were excluded for missing MRI scans, 18 for late MRI scans, 3 for absence of HIE diagnosis in the neurology consult notes, 18 for not undergoing cooling, 4 for being transferred from an outside facility later than day of life 1, and 1 for being under 35 weeks gestation. The demographic and clinical variables collected for 123 neonates from the chart review are detailed in Table 1 and seizure characteristics detailed in Table 2. Seizures were present in 39 (31.7%) neonates. All but three had confirmed electrographic seizures once placed on an EEG. Neonates with MRI changes in the splenium had higher rate of seizure present, higher seizure burden, mortality during hospitalization, proportion requiring intubations and proportion with low APGAR score at 10 min.

**Table 1:** Demographic and clinical characteristics of neonates diagnosed with HIE and underwent cooling

	Total (*N* = 123)	MRI change in splenium		
		Yes (*N* = 26)	No (*N* = 97)	*P*
Gestation (weeks)	39.16 ± 1.60	39.60 ± 1.48	39.05 ± 1.62	0.120
Preterm < 37 weeks gestation^a^	15 (12.3%)	3 (11.5%)	12 (12.5%)	0.895
Birth weight (grams)	3199.07 ± 699.65	3224.04 ± 899.76	3192.37 ± 641.13	0.839
Female sex	57 (46.3%)	12 (46.2%)	45 (46.4%)	0.983
C-section	81 (65.9%)	18 (69.2%)	63 (64.9%)	0.683
Non-reassuring fetal heart tone^a^	85 (69.7%)	19 (73.1%)	66 (68.8%)	0.670
Pre-eclampsia^a^	6 (4.9%)	0 (0.0%)	6 (6.3%)	0.191
Placental abruption, uterine rupture or cord prolapse^a^	20 (16.4%)	4 (15.4%)	16 (16.7%)	0.876
Meconium stain^a^	53 (43.4%)	14 (53.8%)	39 (40.6%)	0.228
Nuchal cord^a^	24 (19.7%)	5 (19.2%)	19 (19.8%)	0.949
Maternal chorioamnionitis^a^	12 (9.8%)	4 (15.4%)	8 (8.3%)	0.284
Intubation	84 (68.3%)	22 (84.6%)	62 (63.9%)	**< 0.05**
Chest compressions	37 (30.1%)	10 (38.5%)	27 (27.8%)	0.294
Day of life MRI completed	5.99 ± 1.13	6.38 ± 1.63	5.89 ± 0.93	**< 0.05**
Mortality during hospitalization	7 (5.7%)	6 (23.1%)	1 (1.0%)	**< 0.001**
Lowest blood pH < 7.1	100 (81.3%)	22 (84.6%)	78 (80.4%)	0.625
Apgar 1 min ≤ 5^a^	118 (96.7%)	24 (92.3%)	94 (97.9%)	0.154
Apgar 5 min ≤ 5^a^	86 (70.5%)	21 (80.08%)	65 (67.7%)	0.195
Apgar 10 min ≤ 5^b^	42 (37.8%)	14 (60.9%)	28 (31.8%)	**< 0.05**
Sarnat staging				
Mild	10 (8.1%)	2 (7.7%)	8 (8.2%)	0.135
Moderate	95 (77.2%)	17 (65.4%)	78 (80.4%)	
Severe	18 (14.6%)	7 (26.9%)	11 (11.3%)	

Data presented as mean ± standard deviation or counts (and percentages within each row). *P*-value for unpaired t-test for continuous variables or Chi-square analysis for categorical variables

^a^One patient with no prenatal care, unknown gestational age, and delivery at home; *N* = 122 for total neonates, *N* = 96 for no splenium change group

^b^Twelve neonates did not have APGAR obtained at 10 min; *N* = 111 for total neonates, *N* = 23 for yes splenium change group, *N* = 88 for no splenium change group

**Table 2:** Seizure characteristics in relation to splenial changes on MRI

	Total (*N* = 123)	MRI change in splenium		
		Yes (*N* = 26)	No (*N* = 97)	*P*
Seizure present	39 (31.7%)	21 (80.8%)	18 (18.6%)	**< 0.001**
Number of anti-epileptics	0.54 ± 1.03	1.77 ± 1.56	0.21 ± 0.46	**< 0.001**
Anti-epileptics used				
None	84 (68.3%)	5 (19.2%)	79 (81.4%)	**< 0.001**
1 anti-epileptic	27 (22.0%)	10 (38.5%)	17 (17.5%)	
2 or more anti-epileptics, not including midazolam drip	7 (5.7%)	6 (23.1%)	1 (1.0%)	
Includes midazolam drip	5 (4.1%)	5 (19.2%)	0 (0.0%)	
Seizure frequency^a^				
None	84 (68.9%)	5 (20.0%)	79 (81.4%)	**< 0.001**
1 seizure	8 (6.6%)	2 (8.0%)	6 (6.2%)	
2 or more seizures	22 (18.0%)	10 (40.0%)	12 (12.4%)	
Status epilepticus	8 (6.6%)	8 (32.0%)	0 (0.0%)	
Worst EEG background in first 24 hours^b^				
Normal	12 (9.8%)	1 (3.8%)	11 (11.5%)	**< 0.001**
Mild to moderate	90 (73.8%)	13 (50.0%)	77 (80.2%)	
Severe	20 (16.4%)	12 (46.2%)	8 (8.3%)	
Seizure type^c^				
Clinical only	3 (7.7%)	2 (9.5%)	1 (5.6%)	0.375
Electrographic only	17 (43.6%)	7 (33.3%)	10 (55.6%)	
Clinical and electrographic	19 (48.7%)	12 (57.1%)	7 (38.9%)	

Data presented as mean ± standard deviation or counts (and percentages within each row). *P*-value for unpaired t-test for continuous variables or Chi-square analysis for categorical variables

^a^One patient had unclear duration of clinical seizures at outside hospital prior to transfer to Rady’s Children’s Hospital; *N* = 122 for total neonates, *N* = 25 for yes splenium change group

^b^One patient had video EEG done but information on EEG background in the first 24 hours was not available; *N* = 122 for total neonates, *N* = 96 for no splenium change group

^c^Eighty-four neonates had no seizures; *N* = 39 for total neonates, *N* = 21 for yes splenium change group, *N* = 18 for no splenium change group

MRI imaging was abnormal in 50 neonates (40.7%), with the involved brain areas summarized in Table 3. A total of 26 (21.1%) neonates had splenium signal abnormality. Abnormal signal in the splenium had the highest specificity and PPV for seizure compared to other brain areas. Examining whether pH or APGAR score contributed to MRI abnormality showed a significant association between APGAR score at 10 min and multiple MRI areas (splenium, cortex, and deep gray matter), and pH and subcortical white matter (Table 4). The presence of seizure was significantly related to brain injury in all the areas studied. A total of 36 neonates (31.0%) followed up with the high-risk infant clinic between 12 to 18 months old and only 24 neonates (19.5%) followed up at > 18 months. Seizure and splenium signal abnormality were significantly associated with developmental delay at 12 to 18 months old (Table 5), but not at > 18 months old (Table 6). In contrast, APGAR score and pH were not significantly related to developmental outcome.

**Table 3:** Performance of various MRI areas on screening for recent seizure in neonates with HIE

Areas	Number (%) of positive MRIs	Sensitivity	Specificity	PPV	NPV	Positive LR	Negative LR
Splenium	26 (21.1%)	53.85%	94.05%	80.77%	81.44%	9.05	0.49
Cortex	34 (27.6%)	58.97%	86.90%	67.65%	82.02%	4.50	0.47
Deep gray matter	22 (17.9%)	41.03%	92.86%	72.73%	77.23%	5.74	0.64
Subcortical white matter	27 (22.0%)	43.59%	88.10%	62.96%	77.08%	3.66	0.64
Any change	50 (40.7%)	69.23%	72.62%	54.00%	83.56%	2.53	0.42

*PPV* Positive predictive value, *NPV* Negative predictive value, *LR* Likelihood ratio

**Table 4:** Chi-square statistics for seizure, blood pH, and APGAR score in relation to various MRI areas

	Seizure	Lowest pH < 7.1	APGAR ≤5 at 5 min	APGAR ≤5 at 10 min
Splenium	36.649, ***P*** **< 0.001**	0.238, N.S.	1.678, N.S.	6.543, ***P*** **< 0.05**
Cortex	28.029, ***P*** **< 0.001**	0.493, N.S.	1.497, N.S.	8.585, ***P*** **< 0.01**
Deep gray matter	26.819, ***P*** **< 0.001**	0.005, N.S.	3.251, N.S.	7.652, ***P*** **< 0.01**
Subcortical white matter	15.607, ***P*** **< 0.001**	5.117, ***P*** **< 0.05**	0.885, N.S.	0.832, N.S.
Any change	19.335, ***P*** **< 0.001**	0.027, N.S.	1.962, N.S.	6.458, ***P*** **< 0.05**

*N.S.* Not significant

**Table 5:** Effect of splenial signal abnormality, seizure, APGAR score, or pH on developmental delay using BSID scale at 12 to 18 months of age

	Normal/no delays (*N* = 21)	Moderate delays (*N* = 11)	Severe delays (*N* = 4)	*P*
Splenium signal abnormality				
Yes	3 (42.9%)	1 (14.3%)	3 (42.9%)	**< 0.05**
No	18 (62.1%)	10 (34.5%)	1 (3.4%)	
Seizure				
Yes	5 (50.0%)	1 (10.0%)	4 (40.0%)	**< 0.01**
No	16 (61.5%)	10 (38.5%)	0 (0.0%)	
Apgar 5 min				
≤ 5	15 (55.6%)	8 (29.6%)	4 (14.8%)	0.471
> 5	6 (66.7%)	3 (33.3%)	0 (0.0%)	
Apgar 10 min				
≤ 5	7 (58.3%)	3 (25.0%)	2 (16.7%)	0.830
> 5	13 (61.9%)	6 (28.6%)	2 (9.5%)	
Lowest blood pH				
< 7.1	19 (59.4%)	9 (28.1%)	4 (12.5%)	1.110
≥ 7.1	2 (50.0%)	2 (50.0%)	0 (0.0%)	

Data presented as counts (and percentages within each row). *P*-value for Chi-square analysis

**Table 6:** Effect of splenial signal abnormality, seizure, APGAR score, or pH on developmental delay using BSID scale at > 18 months to 36 months of age

	Normal/no delays (*N* = 9)	Moderate delays (*N* = 2)	Severe delays (*N* = 3)	*P*
Splenium signal abnormality				
Yes	2 (66.7%)	1 (33.3%)	0 (0.0%)	0.438
No	16 (76.2%)	2 (9.5%)	3 (14.3%)	
Seizure				
Yes	6 (75.0%)	1 (12.5%)	1 (12.5%)	1.000
No	12 (75.0%)	2 (12.5%)	2 (12.5%)	
Apgar 5 min				
≤ 5	14 (70.0%)	3 (15.0%)	3 (15.0%)	0.449
> 5	4 (100.0%)	0 (0.0%)	0 (0.0%)	
Apgar 10 min				
≤ 5	9 (81.8%)	1 (9.1%)	1 (9.1%)	0.777
> 5	9 (69.2%)	2 (15.4%)	2 (15.4%)	
Lowest blood pH				
< 7.1	18 (75.0%)	3 (12.5%)	3 (12.5%)	0.578
≥ 7.1	4 (80.0%)	0 (0.0%)	1 (20.0%)	

Data presented as counts (and percentages within each row). *P*-value for Chi-square analysis

## Discussion

To the best of our knowledge, the present study is the largest series to date on splenium signal abnormality. Our data indicates that decreased diffusivity in the callosal splenium has high specificity and PPV for recent seizures. This may impact the degree of developmental monitoring needed and the need for further testing.

In addition to seizures, our findings are in agreement with previous reports that splenium signal abnormality is associated with more severe clinical presentations and adverse outcomes [12–15]. Perlman and colleagues reported 10 (29%) of their neonates with HIE had restricted diffusion in the splenium [14]. All these patients had clinical or electrographic seizures. They found that those with splenial signal abnormality had a significantly higher incidence of death or severe developmental delay, lower birth weight, lower cord arterial base deficit, and severe encephalopathy during initiation of hypothermia. We similarly found a higher incidence of mortality and a higher proportion with severe developmental delay at 12 to 18 months of age. Moreover, diffusion abnormality was associated with lower APGAR score at 10 min, higher mortality, higher proportion requiring intubation during the first few minutes of life, and greater seizure burden. Kelkar and colleagues reported on 16 (40%) neonates with different patterns of corpus callosum involvement, of which 15 had splenium changes [13]. Kumar et al. found 11 (40%) of their patients had restricted diffusion within the corpus callosum, all of whom had splenium changes [15]. Both of these studies found that corpus callosum injury was associated with more extensive brain injury [13, 15]. We similarly found 69.2% of our neonates with restricted diffusion in the splenium had at least 2 or more other brain regions affected (data not shown). Notably, 100% (18/18) of the neonates with 2 or more brain regions plus splenium change had seizures, whereas only 16.7% (1/6) of the neonates with 2 or more brain regions affected and no splenium change had seizures (Χ^2^ = 18.947, *P* < 0.001), reiterating that splenium change, not diffuse brain injury per se, is significantly associated with recent seizures.

Several possible causes of transient splenium signal abnormality have been reported in the literature. These include acute seizures, anticonvulsant drug withdrawal or toxicity, viral encephalitis, hypoglycemic encephalopathy, malnutrition, traumatic axonal injury, and early Wallerian degeneration [2, 3, 16–19]. Specific to neonates with seizures, there have been isolated case reports, including neonates without HIE [7], mild HIE [9], and hypoglycemia [8]. It is unclear in our cohort whether the splenium changes are transient or permanent. It also remains unknown if this is associated with an increased risk for future seizures. Five neonates had abnormal signal in the splenium without a history of clinical or subclinical seizures. It is possible subclinical seizures occurred between birth and the time EEG was started in these patients, or the neonates had one of the other possible causes of transient splenium signal abnormality. Longer follow-up period and inclusion of all neonates with splenial lesions might clarify these findings in the future.

The underlying pathophysiological mechanism of splenium changes in seizures remains obscure. There have been several hypotheses have been put forward, including cytotoxic, vasogenic, and/or intramyelinic edema [5, 6, 18, 20]. While cytotoxic or vasogenic edema may be relevant in neonates, intramyelinic edema is unlikely a contributing mechanism as the splenium does not begin myelination until 3 months of age.

Our study has several limitations. First, it follows a retrospective design. Second, previous studies have described pseudo-normalization of diffusion weighted imaging and apparent diffusion coefficient maps in neonates with HIE as early as 4 days after the insult and suggested that neonates who are scanned after the first week of life may exhibit false-negative results [21]. We included MRIs obtained up to 10 days of life, which is on the later end of the optimal time window [11]. However, this is unlikely to change our outcome because our MRIs were obtained on an average of 6 days of life, and the splenial abnormality group had scans done on average at a higher day of life than the no splenial abnormality group. Using MRIs obtained up to 10 days of life also improves ecological validity. A third limitation is that only about a third of our patients followed up in the high-risk infant clinic at 12 to 18 months old and even less followed up at > 18 months old, creating a possible selection bias. We however found no significant association between any of the demographic or clinical variables and follow up (data not shown). Only 7 (26.9%) and 3 (11.5%) of the patients with splenium abnormalities had developmental testing at 12 to 18 months old and > 18 months old, respectively, making it difficult to draw any firm correlations based on this reduced sample size. This reduced sample size may also explain why there was a significant association between splenial lesion/seizure and poor developmental outcome at 12 to 18 months old but not at > 18 months old. Fourth, most of our infants studied are relatively young, and long-term neurodevelopmental outcome may not be apparent at the 12 to 18 months visit. For those who had repeat BSID-III testing at > 18 to 36 months old, 10 (62.5%) remained in the same severity classification and 6 (37.5%) had either an improvement or worsening. Finally, we selected neonates with HIE who underwent therapeutic cooling to remove etiology as a confounder for splenium change. Further studies will need to be carried out to determine whether our findings are generalizable to neonates with seizures due to other causes, such as infection, hypoglycemia, cerebral hemorrhage, metabolic disorders, or genetic epilepsies.

## Conclusion

Restricted diffusion of the callosal splenium is specific for recent seizures in neonates with HIE who underwent therapeutic cooling. Splenium lesions are associated with more severe clinical presentations and worst developmental outcomes at 12 to 18 months old. Future studies would be beneficial to determine the relationship between changes in the splenium and long-term seizure and neurodevelopment outcomes.

## Data Availability

The datasets used and/or analyzed during the current study are available from the corresponding author on reasonable request.

## Abbreviations

CC: Corpus callosum
MRI: Magnetic resonance imaging
HIE: Hypoxic ischemic encephalopathy
EEG: Electroencephalogram
NICU: Neonatal intensive care unit
RCH: Rady Children’s Hospital
BSID: Bayley Scales of Infant Development
